# Seed/catalyst-free growth of zinc oxide on graphene by thermal evaporation: effects of substrate inclination angles and graphene thicknesses

**DOI:** 10.1186/s11671-014-0716-z

**Published:** 2015-01-22

**Authors:** Nurul Fariha Ahmad, Kanji Yasui, Abdul Manaf Hashim

**Affiliations:** Malaysia-Japan International Institute of Technology, Universiti Teknologi Malaysia, Jalan Sultan Yahya Petra, 54100 Kuala Lumpur, Malaysia; Department of Electrical Engineering, Nagaoka University of Technology, Kamitomioka-machi, Nagaoka, Niigata 940-2137 Japan

**Keywords:** Graphene, Thermal evaporation, Zinc oxide, Nanorod, Nanocluster, Hybrid integration

## Abstract

A seed/catalyst-free growth of ZnO on graphene by thermal evaporation of Zn in the presence of O_2_ gas was further studied. The effects of substrate positions and graphene thicknesses on the morphological, structural, and optical properties were found to be very pronounced. By setting the substrate to be inclined at 90°, the growth of ZnO nanostructures, namely, nanoclusters and nanorods, on single-layer (SL) graphene was successfully realized at temperatures of 600°C and 800°C, respectively. For the growth on multilayer (ML) graphene at 600°C with an inclination angle of 90°, the grown structures show extremely thick and continuous cluster structures as compared to the growth with substrate’s inclination angle of 45°. Moreover, the base of nanorod structures grown at 800°C with an inclination angle of 90° also become thicker as compared to 45°, even though their densities and aspect ratios were almost unchanged. Photoluminescence (PL) spectra of the grown ZnO structures were composed of the UV emission (378–386 nm) and the visible emission (517–550 nm), and the intensity ratio of the former emission (*I*_UV_) to the latter emission (*I*_VIS_) changed, depending on the temperature. The structures grown at a low temperature of 600°C show the highest value of *I*_UV_/*I*_VIS_ of 16.2, which is almost two times higher than the structures grown on SL graphene, indicating fewer structural defects. The possible growth mechanism was proposed and described which considered both the nucleation and oxidation processes. From the results obtained, it can be concluded that temperature below 800°C, substrate position inclined at 90° towards the gas flow, and ML graphene seems to be preferable parameters for the growth of ZnO structures by thermal evaporation because these factors can be used to overcome the problem of graphene’s oxidation that takes place during the growth.

## Background

In recent years, inorganic semiconductor nanostructures and thin films on graphene is particularly interesting because these structures can offer additional functionality to graphene for realizing advanced electronic and optoelectronic applications in photovoltaics, nanogenerators, field emission devices, sensitive biological and chemical sensors, and efficient energy conversion and storage devices [1-13]. Graphene has a great potential for novel electronic devices because of its extraordinary electrical, thermal, and mechanical properties, including a carrier mobility exceeding 10^4^ cm^2^/Vs and a thermal conductivity of 10^3^ W/mK [14-18]. Therefore, with the excellent electrical and thermal characteristics of graphene layers, growing inorganic semiconductor nanostructures and thin films on graphene layers would enable their novel physical properties to be exploited in diverse sophisticated device applications [19,20]. It is worth noting that the atomic arrangement of graphene is similar to the (111) plane of zincblende structure and *c*-plane of a hexagonal crystalline structure which makes the growth of semiconductor nanostructures and thin film on graphene feasible.

Since a decade ago, intensive researches have been focused on fabricating one-dimensional (1D) zinc oxide (ZnO) semiconducting nanostructures because it can provide a variety of important applications due to their unique morphologies, compositions, and chemical/physical properties [21,22]. For example, since graphene is an excellent conductor and transparent material, the hybrid structure of ZnO/graphene shall lead to several device applications not only on silicon (Si) substrate but also on other arbitrary substrates such as transparent glass [8] and transparent flexible plastic [16]. Owing to the unique electronic and optical properties of ZnO nanostructures, such hybrid structure can be used for sensing devices [23-25], ultraviolet (UV) photodetectors [26], solar cells [27], light-emitting diodes [28], etc.

The most common method to grow ZnO on graphene is thermal evaporation due to its simplicity and high growth rates. Recently, we reported the seed/catalyst-free growth of ZnO on multilayer (ML) graphene by thermal evaporation of Zn in the presence of oxygen (O_2_) gas [3]. The effects of substrate temperatures were studied where it was found that the changes of morphologies were very significant. The grown ZnO structures show three different structures, i.e., nanoclusters, nanorods, and thin films at 600°C, 800°C, and 1,000°C, respectively. High-density vertically aligned ZnO nanorods comparable to other methods were also obtained. However, two important issues still remain. First, the growth mechanism of nanostructures has to be investigated, which would increase controllability in the morphology and chemical and physical properties of nanostructures. Second, uniform and homogeneous growth of nanostructures on large graphene films would be very crucial for the practical use. By considering these two issues, the effects of graphene thickness and substrate position have been set to be further studied in this paper. As reported by Lu et al., the inclination angle of the substrate was shown to give significant effects in terms of the density and length of the grown ZnO nanowires on Si due to the differences of incoming gas flow patterns. It is worth noting that the growth technique and setup are similar to our work. Motivated by this point, in this work, we chose to test two kinds of inclination angles which are 45° and 90° as we expect that the significant differences should be observable especially for the growth of ZnO on graphene [29].

## Methods

**Figure 1:**
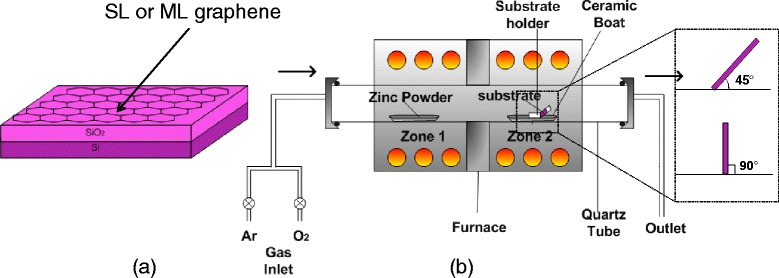
**Schematic of (a) graphene/SiO**_**2**_ **/Si substrate and (b) growth setup.**

Two kinds of graphene structures, i.e., single-layer (SL) graphene and ML graphene, grown by chemical vapor deposition (CVD) and transferred onto silicon dioxide (SiO_2_)/Si were used as the substrates (Graphene Laboratories, USA). Figure 1a shows the schematic of graphene layer on 285-nm-thick SiO_2_. The growth was carried out by thermal evaporation technique in dual zone furnace as shown in Figure 1b. A high purity of metallic Zn powder (99.85%) and oxygen (O_2_) gas (99.80%) were used as the sources. Prior to the growth process, the substrate was treated with organic cleaning of ethanol, acetone, and deionized (DI) water to remove any unwanted impurity on the substrate. Zn powder of approximately 0.6 g was spread evenly into a ceramic boat. The ceramic boat was placed in the zone 1 of furnace while the substrate was placed with inclination angles of 45° and 90° facing towards the flow of O_2_ gas in the zone 2 of furnace. The distance between source and substrate was fixed at 23 cm. This is a distance from center-to-center of two zones. Two independent temperatures were applied to the furnace system. Here, T1 denotes to the set temperature (ST) of the source while T2 denotes to the ST of the substrate. Firstly, the temperature of zone 2 was raised to T2 (i.e., 600°C, 800°C, or 1,000°C) in argon (Ar) environment (Ar flow rate of 200 sccm). Then, the temperature of zone 1 was raised to T1 (1,000°C). The flow of Ar was stopped when the temperature of zone 1 reached 400°C (Zn melting point, 419°C). This was done in order to avoid the transfer of Zn particles to substrate prior to actual growth. The heating of Zn powder was continued until it reached 1,000°C. It was confirmed from several attempts that such high temperature of 1,000°C was needed for continuous and constant evaporation of Zn. After reaching 1,000°C, O_2_ (400 sccm) was introduced for 1 h of growth time. Finally, the furnace was turned off and the samples were cooled down to room temperature. It is noted here that the above procedures are identical with the procedures reported in ref. [3]. The as-grown ZnO were examined using field-emission scanning electron microscopy (FESEM) (Hitachi SU8030), energy-dispersive X-ray (EDX) spectroscopy, X-ray diffraction (XRD) (Bruker, AXES, D8 Advance), and photoluminescent (PL) spectroscopy (Horiba Jobin Yvon).

## Results and discussion

**Figure 2:**
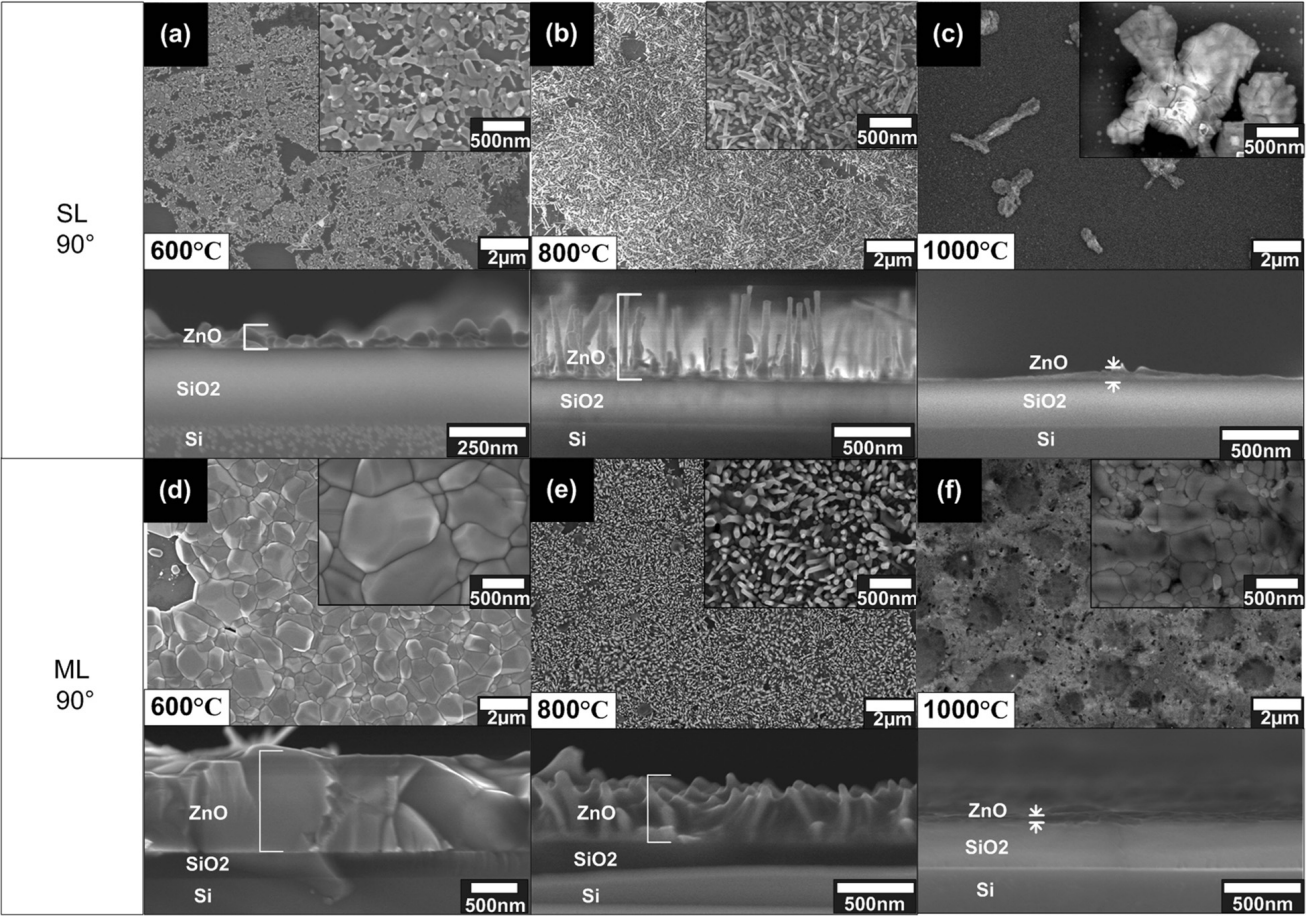
**FESEM images of the grown ZnO on SL and ML graphene with substrate’s inclination angle of 90°. (a)** SL 600°C, **(b)** SL 800°C, **(c)** SL 1,000°C, **(d)** ML 600°C, **(e)** ML 800°C, and **(f)** ML 1,000°C.

It is worth noting that the growth on SL graphene with a substrate’s inclination angle of 45° was not obtained in our previous work [3]. It is speculated that since the thickness of a SL graphene is only one monolayer, the oxidation or etching rate of SL graphene is considerably remarkable as compared to ML graphene. Also, the small nucleation rate probably due the substrate position which is placed 45° inclined to the flow of O_2_ gas. In order to realize the growth of ZnO structure on SL graphene, the nucleation rate should be increased. It is expected that the change of the substrate’s inclination angle to 90° may help to realize such situation. Figure 2 summarizes the observed surface morphologies of the grown ZnO structures on SL and ML graphene with substrate’s inclination angle of 90°. Figure 2a–c shows the results of the growth on SL graphene at temperatures of 600°C, 800°C, and 1,000°C, respectively. As shown in Figure 2a, it can be clearly seen that non-continuous nanocluster-like structures were grown at a temperature of 600°C. Meanwhile, a mixture of vertically non-aligned and aligned nanorods was grown at a temperature of 800°C. It is worth noting that for the growth at 800°C as shown in Figure 2b. Moreover, several randomly dispersed microcluster-like structures were observed for the growth at a temperature of 1,000°C as shown in Figure 2c. Here, it can be said that the growth of ZnO structures was almost unachievable at 1,000°C for the growth on SL graphene due to the possible severe oxidation of graphene during the growth [3]. It was also confirmed by the EDX measurement that no carbon (C) element was detected at the area without ZnO structures. From these results, it can be simply concluded that the nucleation of ZnO was achieved at an inclination angle of 90° for the growth on SL graphene. Figure 2d–f shows the results of the growth on ML graphene with an inclination angle of 90° at temperatures of 600°C, 800°C, and 1,000°C, respectively. As reported in our previous work [3], the grown ZnO structures on ML graphene with an inclination angle of 45° show three different structures, i.e., non-continuous nanoclusters, nanorods, and thin films at 600°C, 800°C, and 1,000°C, respectively. In principle, such similar basic structures were also observed for an inclination angle of 90°, as shown in the corresponding Figure 2d–f. As shown in Figure 2d, it can be clearly seen that continuous ZnO clusters with larger grain size and thicker layer structures than those of structures grown with an inclination angle of 45° [3] were obtained. As shown in Figure 2e, nanorods with similar morphological structures (see Table 1) was obtained for the growth at a substrate temperature of 800°C as compared to nanorods grown at the same temperature with an inclination angle of 45° [3]. However, as shown in Figure 2f, the morphologies of grown structures at 1,000°C with an inclination angle of 90° do not show significant difference with the structure grown at the same temperature with an inclination angle of 45° [3] where the structures were in the form of thin film structures. From the growth on ML graphenes at both inclination angles, it can be concluded that the basic structures of ZnO strongly depend on the substrate temperatures. Based on these results, it also seems to simply indicate that an inclination angle of 90° tends to increase or promote the nucleation of ZnO structures. It is reported that the nucleation of ZnO on graphene is generally achieved at the locations of “etch pit” where the breaking of C-C bonds occurs [3]. The breaking of C-C bonds of graphene is realized by the oxidation process that also takes place during the growth. Besides that, the nucleation of ZnO on ML graphene is further promoted as compared to SL graphene likely due to the stacking structure of ML graphene where graphene edges provide extra nucleation sites for the ZnO growth [5]. Therefore, based on the obtained results, it can be concluded that not only the substrate temperature but also the substrate position and graphene thickness give significant influences in the growth of ZnO structures.

**Table 1:** **Morphology, density, diameter, length, thickness, and average aspect ratio of the grown ZnO structures**

**Substrate**	**Substrate position**	**Temperature (°C)**	**Morphology**	**Density (cm** ^**−2**^ **)**	**Diameter of nanorods/nanoneedles (nm)**	**Length of nanorods (nm)**	**Thickness (nm)**	**Average aspect ratio**
SL graphene on SiO_2_/Si	90° inclined	600	Non-continuous nanoclusters	-	-	-	~93	-
This work	90° inclined	800	A mixture of vertically non-aligned and aligned nanorods	1.2 × 10^9^	37–74	93–574	~40 (base structure)	~5.1
	90° inclined	1,000	Randomly dispersed clusters	-	-	-	-	-
ML graphene on SiO_2_/ Si	90° inclined	600	Continuous clusters/layer	-	-	-	~1,062	-
This work	90° inclined	800	Nanorods	7.0 × 10^9^	<150	<540	~90 (base structure)	~2.8
	90° inclined	1,000	Thin film	-	-	-	~74	-
ML graphene SiO_2_/ Si by thermal evaporation [3]	45° inclined	600	Non-continuous nanoclusters	-	-	-	~200	-
	45° inclined	800	Nanorods	6.9 × 10^9^	<150	<540	~50 (base structure)	~2.8
	45° inclined	1,000	Thin film	-	-	-	~60	-
Porous silicon by thermal evaporation [21]	Placed on source boat and facing downward	800	Nanorods	1.2 × 10^8^	200–500	Not stated	-	Not stated
ML graphene on SiO_2_/Si by MOVPE [1]	Standard horizontal position	400	Nanorods	4.0 × 10^9^	100 ± 10	1,000 ± 100	-	~10.0
		600	Nanoneedles	8.0 × 10^9^	90 ± 20	4,000 ± 600	-	~44.4
		750	Nanoneedles	5.0 × 10^7^	Not stated	3,500 ± 500	-	Not stated

Table 1 summarizes the morphology, density, diameter, length, thickness, and average aspect ratio of the grown ZnO structures including comparison with other works [1,3,21]. In general, the densities of the grown nanorods are comparable or even larger than the densities of nanorods grown by other techniques. For the growth at a low temperature of 600°C, the thickness of deposited ZnO continuous cluster structure on ML graphene with a substrate’s inclination angle of 90° is found to be five times larger than the thickness of ZnO nanocluster structure grown at the substrate’s inclination angle of 45°. The density of vertically aligned ZnO nanorods on SL graphene grown at a temperature of 800°C is estimated to be around 1.2 × 10^9^ which is much lesser than ML graphene. The calculated densities of vertically aligned nanorods for the sample grown on ML graphene at 800°C with an inclination angle of 90° is estimated to be around 7.0 × 10^9^ which is almost identical to the density of nanorods grown on ML graphene [3] at the same temperature with an inclination angle of 45°. From the cross-sectional images, the length and diameter of the nanorods were measured, and then, the calculated value of average aspect ratios was determined. The average aspect ratio for nanorods grown on ML graphene at both inclination angles shows the same value which is 2.8. It was found that the aspect ratio for ZnO nanorods grown on SL graphene at the same temperature is almost two times larger than that of nanorods grown on ML graphene. As mentioned, it is speculated that since there is no additional nucleation sites in SL graphene as compared to ML graphene, the subsequent nucleation and growth of ZnO seems to be less and limited. The reason of low aspect ratio for nanorods grown on ML graphene may be due to the larger initial nucleations at the stacking area, resulting to the subsequently grown nanorod diameter to be large. This mechanism is elaborated in the last section of this article.

**Figure 3:**
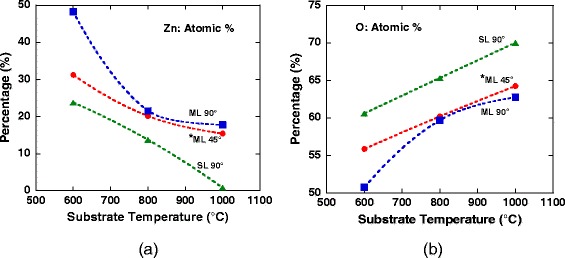
**Atomic percentage of (a) Zn and (b) O of grown ZnO on SL and ML graphene at substrate’s inclination angle of 45° and 90°.** Asterisk indicates that the data are extracted from the work presented in ref. [3].

Figure 3a,b shows the measured atomic percentage of Zn and O, respectively. The percentages of Zn composition in the grown structure decrease with the temperatures due to the lower density of nucleated ZnO at high temperature, resulting to the opposite tendency in O percentages. It is worth noting that the percentage of detected O element at high temperature is basically contributed by the O element from the SiO_2_ layer. This tendency is consistent with the FESEM results as shown in Figure 2. Here, it also seems to prove the hypothesis that the nucleation rate of Zn is much higher on ML graphene than that of on SL graphene by considering the high percentage of Zn composition in ML graphene. Also, the nucleation rate at an inclination angle of 90° is higher than that of 45° especially for the growth temperature of 600°C by comparing the grown structures on ML graphene. Table 2 summarizes the compositional ratio of Zn atoms to O atoms in the grown samples. A considerably high ratio value of 0.95 was obtained for the structures grown on ML graphene at 600°C with an inclination angle of 90°. The values for the structures grown on ML graphene at 800°C and 1,000°C grown with substrate’s inclination angle of 90° is slightly higher than the structures grown with substrate’s inclination angle of 45°. In general, the ratio is small for the structures grown on SL graphene. However, the value for the structures grown at 600°C on SL graphene was found to be slightly higher than the values of structures grown on ML graphene at temperatures of 800°C and 1,000°C, suggesting that temperature is also an effective control parameter in determining the nucleation rate. This result shows that the nucleation of Zn particles is less promoted at high temperatures and also on SL graphene. It is speculated that such tendency may be due to the severe oxidation of graphene during the growth resulting to the formation of large etch pit [3] with the increase of temperature as well as less nucleation sites on SL graphene as compared to ML graphene.

**Table 2:** **Compositional ratio of Zn atoms to O atoms in the grown samples**

**Temperature (°C)**	**ML graphene**		**SL graphene**
	**Inclination angle of 45°**	**Inclination angle of 90°**	**Inclination angle of 90°**
600	0.55^a^	0.95	0.39
800	0.33^a^	0.36	0.21
1,000	0.23^a^	0.28	0.01

^a^Data extracted from ref. [3].

**Figure 4:**
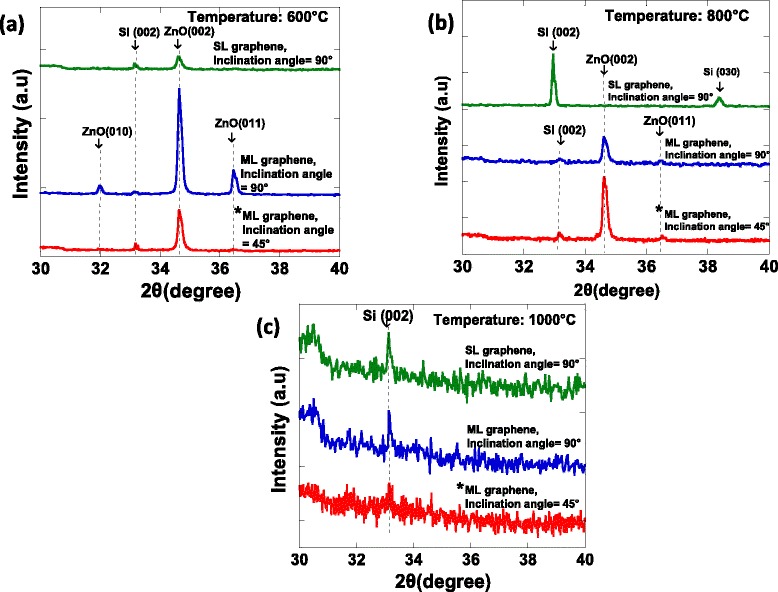
**XRD spectra of grown ZnO structures on SL and ML graphene at (a) 600°C (b) 800°C, and (c) 1,000°C.** Asterisk indicates that the data are extracted from ref. [3].

Figure 4a–c shows the measured XRD spectra for the grown ZnO structures on SL and ML graphene at temperatures of 600°C, 800°C, and 1,000°C, respectively. The as-grown ZnO structures exhibit a hexagonal wurtzite structure as indicated by the presence of prominent peaks at ~34.46°, 32.01°, and 36.51° corresponding to ZnO (002), ZnO (010), and ZnO (011) diffraction peaks, respectively. The relatively high intensity of ZnO (002) peak as compared to other ZnO-related peaks indicates the highly oriented as-grown ZnO in *c*-axis especially for samples grown on ML graphene at temperatures of 600°C and 800°C. It is noted here that the ZnO-related peak was not observed for the ZnO growth on SL graphene at a temperature of 800°C as shown in Figure 4b, probably due to the thin base structure and low density of nanorods. In opposite, the relatively strong peak that corresponded to Si (002) diffraction peak was observed, suggesting the grown structures were very thin to be detected by XRD measurement. This is also evidenced by the FESEM images where the grown structures were basically dominated by the thin non-aligned structures. The structures grown at 1,000°C do not show any peak related to ZnO structures as shown by Figure 4c. This is also most probably due to the thickness of the grown layer was very thin.

**Figure 5:**
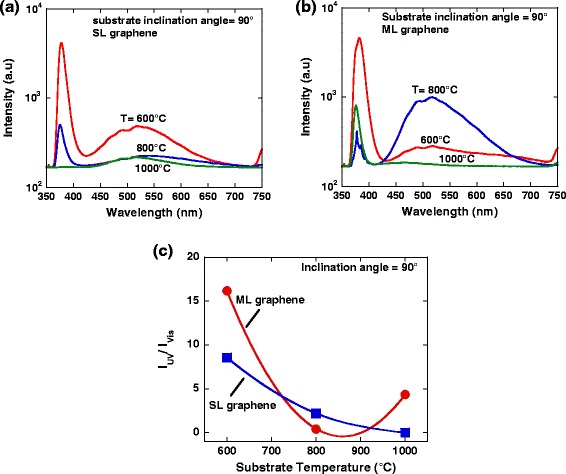
**PL spectra of grown ZnO structures on (a) SL graphene and (b) ML graphene with inclination angle of 90°, and (c) intensity ratio of the peaks in UV region and visible region.**

Figure 5a,b shows the measured PL spectra for the ZnO structures grown on SL and ML graphene with a substrate’s inclination angle of 90°, respectively. It can be seen that, the UV emission in the range of 378–386 nm were detected in the grown samples. It is noted that this emission is an intrinsic property of the wurtzite ZnO which originates from the near-band edge (NBE) or excitonic recombination. It is reported that the enhancement of UV emission is attributed by a larger surface area and fewer defects [30]. Meanwhile, the green emission in the visible region in the range of 517–550 nm was also observed in the grown samples. It is reported that the peak is attributed to specific defects such as O vacancies and Zn interstitials, and these defects are responsible for the recombination of the green luminescence [31,32]. In order to understand the level of structural defects of the grown samples, the intensity ratio of the emission in UV region over the emission in visible region (*I*_UV_/*I*_VIS_) was calculated. Figure 5c shows the calculated intensity ratio of the grown structures. The high value of this ratio indicates fewer structural defects of the nanostructure. It can be seen that the structures grown on both graphene thicknesses show the highest value of intensity ratio at a low temperature of 600°C as compared to other high temperatures, and the structures grown on ML graphene at a temperature of 600°C shows a value of 16.2, which is almost two times higher than the structures on SL graphene at the same temperature. This simply indicates that the structures grown at a low temperature of 600°C contain low structural defects.

**Figure 6:**
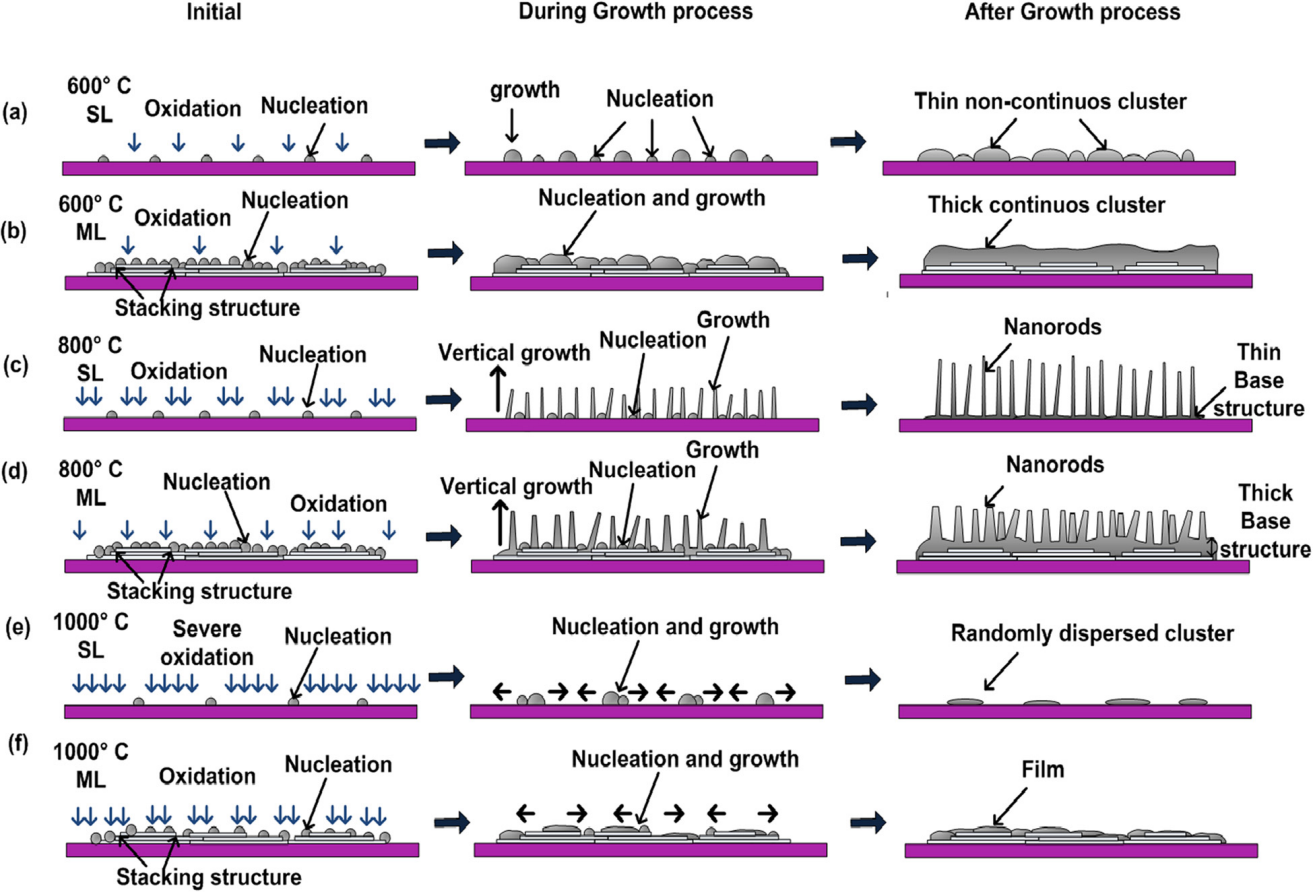
**Possible growth mechanism of ZnO structures. (a)** SL 600°C, **(b)** ML 600°C, **(c)** SL 800°C, **(d)** ML 800°C, **(e)** SL 1,000°C, and **(f)** ML 1,000°C.

As reported in ref. [3], the nucleation rates of ZnO on graphene strongly depend on the breaking rates of C-C bonds of graphene, namely, oxidation, and it takes place as soon as the O_2_ gas is introduced into the system. This oxidation process seems to be faster than the nucleation of Zn particles when the position of substrate is inclined to 45°. This can be proved where no nucleation of ZnO was obtained for the growth on 45°-inclined SL graphene, but the growth was successfully realized on 90°-inclined SL graphene. It is also speculated that 90°-inclined position seems to help in increasing the nucleation rates. As mentioned in the previous section, the nucleation of Zn on ML graphene is also promoted by the availability of graphene stacking structures which provide graphene edges as the additional nucleation sites [5]. This also seems to result in a large nucleation diameter. Hence, the subsequent growth of ZnO will result to large diameters of ZnO continuous clusters and ZnO nanorods grown at temperatures of 600°C and 800°C, respectively. This can be evidenced from the FESEM images shown in Figure 2 and low aspect ratio shown in Table 1. For the case of SL graphene, since the nucleation sites are only provided by the etch pit created by the oxidation process, the numbers of nucleation sites is small and the subsequent growth of ZnO structures will be based on these small nucleation sites, resulting to the thin non-continuous cluster at a temperature of 600°C and small diameter of nanorods at a temperature of 800°C. This can also be evidenced from the FESEM images shown in Figure 2 and high aspect ratio shown in Table 1. It can be also concluded that temperature of 1,000°C is too high, which creates severe oxidation of graphene and limits the nucleation of ZnO. Since the SL graphene is thinner compared to ML graphene, the graphene can be easily etched away at high temperature as compared to ML graphene. As described in ref. [3], the temperature around 800°C is the ideal temperature to promote the vertical growth which will result to the formation of vertically aligned nanorods. Figure 6 illustrated the proposed mechanism and this mechanism is the improved version and agrees with the one proposed in ref. [3].

## Conclusions

In conclusion, the effects of substrate position and graphene thickness on the morphological, structural, and optical properties were found to be very pronounced. Substrate position with an inclination angle of 90° seems to be one of the effective control parameters to increase the nucleation rates which were proven by the ability to grow ZnO nanostructures on SL graphene as well as the growth of thick and continuous ZnO cluster structures and thick base structures of ZnO nanorods on ML graphene. The structures grown at a low temperature of 600°C show fewer structural defects. The possible growth mechanism is proposed and described which considered both the nucleation and oxidation processes. Not only temperature but also substrate position and graphene thickness play significant role in determining the properties of the grown ZnO structures by thermal evaporation because these factors can be exploited to overcome the problem of graphene’s oxidation that takes place during the growth.
